# A Thermally Controlled Multifunctional Metamaterial Absorber with Switchable Wideband Absorption and Transmission at THz Band

**DOI:** 10.3390/ma16020846

**Published:** 2023-01-15

**Authors:** Liansheng Wang, Quanhong Fu, Fusan Wen, Xia Zhou, Xueyong Ding, Yuan Wang

**Affiliations:** 1 Science and Technology Department, Sanya University, Sanya 572022, China; 2 Science Department, Northwestern Polytechnical University, Xi’an 710072, China; 3 Hainan Zhongnanbiao Institute of Quality Science, Sanya 572022, China

**Keywords:** metamaterial absorber, thermally controlled, multifunction, VO_2_, resistive film

## Abstract

This paper proposes a thermally controlled multifunctional metamaterial absorber with switchable wideband absorption and transmission at the THz band based on resistive film and vanadium dioxide (VO_2_). The function of the absorber can be adjusted by changing the phase transition characteristics of VO_2_. When VO_2_ is in a metallic state, the absorber can achieve wideband absorption with above 90% absorption from 3.31 THz to 10 THz and exhibits excellent absorption performance under a wide range of incident and polarization angles. When VO_2_ is in an insulating state, the metamaterial acts in transmission mode with a transmission coefficient of up to 61% at 5.15 THz. The transmission region is inside the absorption band, which is very important for practical applications. It has the advantages of having a simple structure, wideband absorption, and switchable absorption/transmission with potential application value in the fields of stealth of communication equipment and radar at the THz band.

## 1. Introduction

As an artificial composite material, metamaterial at THz band has many unique characteristics that natural materials do not possess, such as super lens [1,2,3], perfect absorption, apparatus [4,5,6,7], invisibility cloak [8,9], etc., this makes it suitable for wide application prospects in many fields. The absorber at the THz band is a device with high absorption to incident electromagnetic waves, which holds a wide application value in wireless security, radar communication, and selective transceivers [10,11,12,13,14,15]. Since it is difficult to realize perfect absorption at the THz band using natural materials, metamaterial absorbers have become a research hotspot in this field. The metamaterial absorber based on electromagnetic resonance was first proposed in 2008, and its absorption at 11.5 GHz was greater than 88%. Later, researchers studied and designed various metamaterial absorbers [16,17,18,19], but their functions are usually fixed, which limits their application prospects. Therefore, the multifunctional metamaterial absorber has a great application value in terahertz systems.

In some fields, such as the security of communication and radar detection, the metamaterial absorber with a transmission window is strongly demanded. It can be realized by using a lossless frequency selective surface instead of the metallic ground plate in the metamaterial absorber structure. In recent years, many kinds of metamaterial absorbers with switchable transmission windows have been proposed [20,21,22,23]. The most reported metamaterial absorbers with switchable transmission windows are based on PIN diodes, but there are few reports depicting other methods to realize it.

VO_2_ thin films have unique insulator−metal phase transition characteristics [24]. When the temperature is about 340 K, the conductivity of VO_2_ will have a significant mutation of five orders and can realize the transition between insulator and metal. Based on this characteristic, VO_2_ thin films have been widely used to design wideband absorbers, absorption–polarization converters, and wideband–narrowband conversion absorbers [25,26,27]. Recently, some researchers use VO_2_ thin film to realize photonic crystal metamaterial−based sensors [28,29,30]. In this paper, we report a thermally controlled multifunctional metamaterial absorber with switchable wideband absorption and transmission at the THz band based on VO_2_. By changing the temperature of VO_2_ thin film to realize the conversion between insulator and metal, the metamaterial absorber can switch between wideband absorption and transmission at the THz band. It expands the application fields of VO_2_ thin film [28,29,30] and has the advantages of having a simple structure, wideband absorption, and switchable absorption/transmission with the potential application value in the fields of stealth of communication equipment and radar at the THz band.

## 2. Model Design

**Figure 1:**
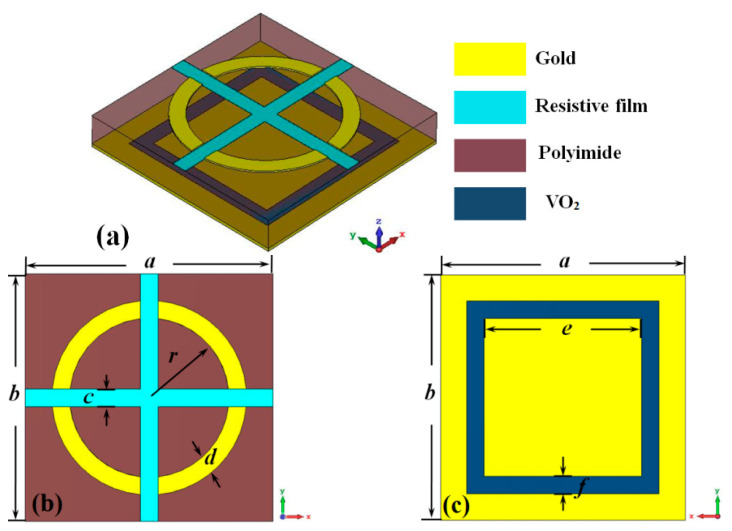
The unit cell of the metamaterial absorber. (**a**) Perspective view; (**b**) front view; and (**c**) back view.

The unit cell of our designed thermally controlled multifunctional metamaterial absorber with switchable wideband absorption and transmission at THz band is shown in Figure 1, which is composed of three layers: the split gold ring loaded with resistive film (conductivity σ=11 S/m) at the top layer, the polyimide (permittivity ε=3.5, the tangent value of loss angle tanδ=0.0027) at the middle layer and the gold ground plate with a square slot, which is filled with VO_2_ film. The thickness of the front and the bottom layer are both 0.02 μm, and the thickness of the middle layer is 2 μm. The optimized structural dimension parameters are *a* = *b* = 14 μm, *r* = 5 μm, *c* = *d* = 1 μm, *e* = 10 μm, and *f* = 1 μm.

At the THz frequency band, the permittivity of VO_2_ film can be expressed by the Drude model:

(1) $$\varepsilon (\omega )=\varepsilon_{\infty }-\frac{\omega_{P}^{2}(\sigma )}{\omega^{2}+i\gamma \omega }$$

In Equation (1), $\varepsilon_{\infty }$ is the high−frequency permittivity with the value of 12, γ is the collision frequency with a value of $5.73\times 10^{13}\;\mathrm{rad}/s$, and $\omega_{p}^{}(\sigma )$ is the plasma frequency, which is related to the conductivity $\sigma_{0}$. The plasma frequency $\omega_{p}^{}(\sigma )$ can be expressed as $\omega_{p}^{2}(\sigma )=(\sigma /\sigma_{0})\omega_{P}^{2}(\sigma_{0})$, wherein $\sigma_{0}=3\times 10^{5}\;S/m$, and $\omega_{P}^{}(\sigma_{0})=14\times 10^{15}\;\mathrm{rad}/s$. In order to simulate the insulator−metal phase transition property of VO_2_, we use $\sigma_{{\mathrm{VO}}_{2}}=2\times 10^{5}\;S/m$ and $\sigma_{{\mathrm{VO}}_{2}}=10\;S/m$, respectively, to represent the metallic state and insulator state of VO_2_ film during the simulation process [31], which can be changed by controlling the temperature of VO_2_ thin film. The different conductivity of VO_2_ can be obtained by different ambient temperatures, and its frequency dependence can be negligible [31]. When the conductivity of VO_2_ film is 2 × 10^5^ S/m, the gold ground plate with a square slot that is filled with VO_2_ film can be seen as a metal ground plate. At this time, the unit cell of the metamaterial absorber can be seen as a circuit resonant structure. The circuit resonance is relatively stable for the change of frequency. Additionally, its surface impedance can match well with the free space in a wide frequency band near the frequency of resonance frequency, which can be used to achieve wideband absorption. When the conductivity of VO_2_ film is 10 S/m, the gold ground plate with the square slot filled with VO_2_ film can be seen as a frequency−selective surface, which can transmit waves at a certain frequency.

The electromagnetic performance of such a metamaterial absorber is numerically studied using the commercial software CST Microwave Studio. During the simulation process, the boundary condition for the *x* direction and *y* direction is set as a unit cell and the boundary condition for the *z* direction is set as open. The All + Floquet modes are used to simulate the incident wave.

## 3. Results and Discuss

**Figure 2:**
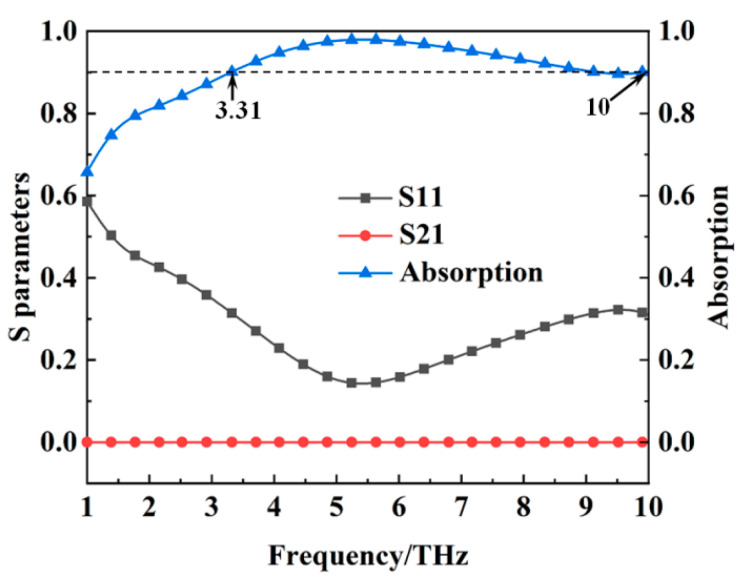
The S parameters and absorption of the metamaterial absorber when the conductivity of VO_2_ film is 2 × 10^5^ S/m.

**Figure 3:**
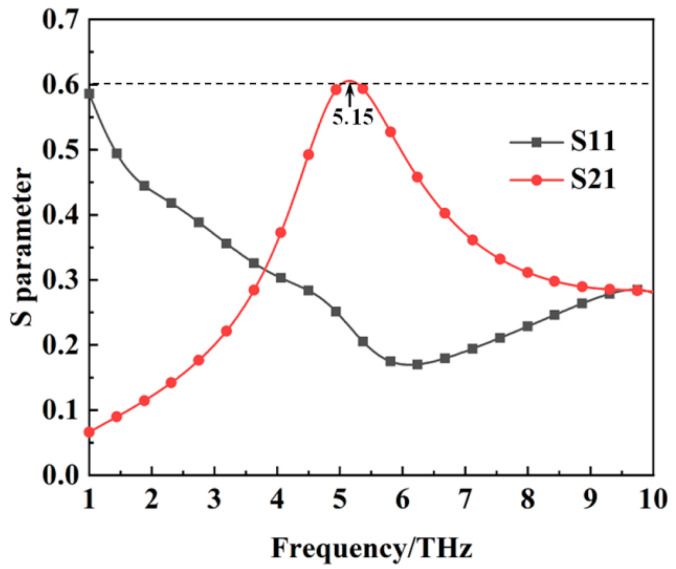
The S parameters of the metamaterial absorber when the conductivity of VO_2_ film is 10 S/m.

The reflection coefficient, transmission coefficient, and absorption of the metamaterial absorber when the conductivity of VO_2_ is 2 × 10^5^ S/m are shown in Figure 2. It can be seen from Figure 2 that the absorption of the metamaterial absorber is more than 90% from 3.31 THz to 10 THz with a relative bandwidth of 100.5%. At this stage, the metamaterial can realize the wideband metamaterial absorber. Since VO_2_ can also act as a metal reflector in the metallic state, the transmission coefficient of the metamaterial absorber tends to be zero. Figure 3 shows the reflection coefficient and transmission coefficient of the metamaterial absorber when the conductivity of VO_2_ is 10 S/m, and we can see that the transmission coefficient is up to 61% at 5.15 THz. The transmission frequency is inside the absorption band, which is very important for practical applications. The above results indicate that the metamaterial absorber has the property of thermally controlled switchability between wideband absorption and transmission at the THz band.

**Figure 4:**
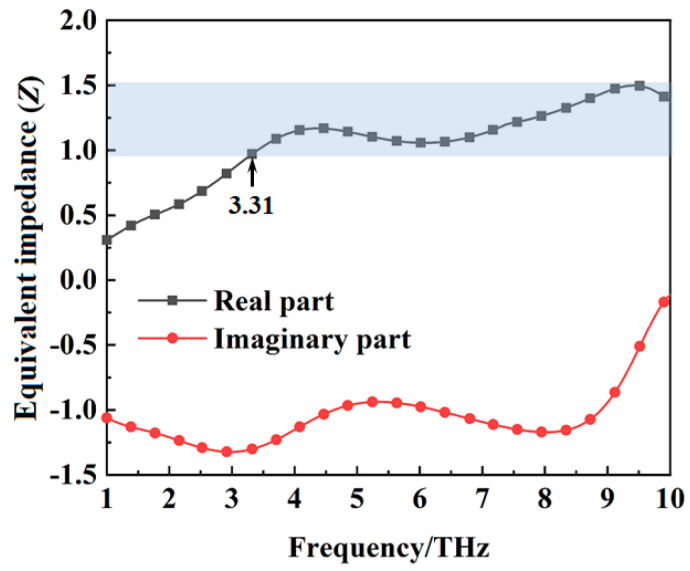
The equivalent impedance of the metamaterial absorber when the conductivity of VO_2_ is 2 × 10^5^ S/m.

**Figure 5:**
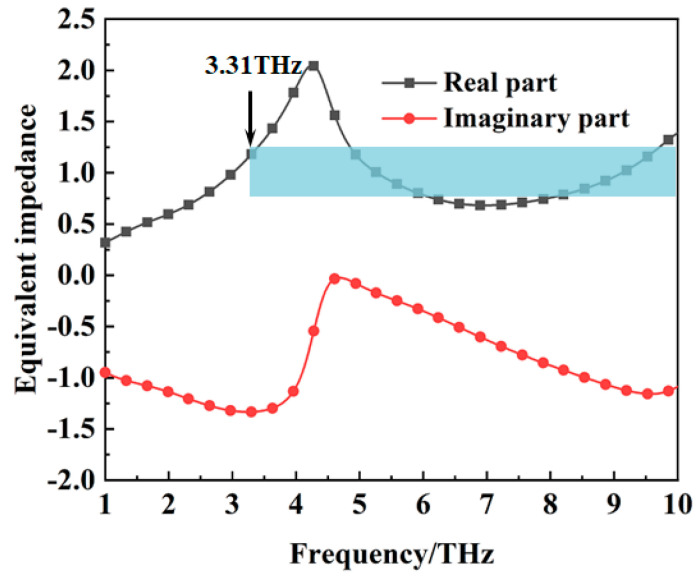
The equivalent impedance of the metamaterial absorber when the conductivity of VO_2_ is 10 S/m.

The wideband absorption mechanism of the metamaterial absorber can be explained by the wave impedance matching principle. For a metamaterial absorber, the equivalent impedance can be expressed as $Z=\sqrt{\frac{{\left(1+S_{11}\right)}^{2}-S_{21}^{2}}{{\left(1-S_{11}\right)}^{2}-S_{21}^{2}}}$ [32]. When the equivalent impedance *Z* = 1, the incident wave can enter the absorber without reflection and be converted into heat energy. According to the *S* parameters obtained from the simulation, the equivalent impedance of the metamaterial absorber when the conductivity of VO_2_ film is 2 × 10^5^ S/m is shown in Figure 4. The real part of the equivalent impedance is nearly equal to one from 3.31 THz to 10 THz. Good impedance matching lays the foundation for wideband absorption of the incident waves by the metamaterial absorber. The equivalent impedance of the metamaterial absorber when the conductivity of VO_2_ film is 10 S/m is shown in Figure 5. The real part of the equivalent impedance is far from one between 3.31 THz to 10 THz. At this time, the gold ground plate with the square slot filled with VO_2_ film can be referred to as a frequency−selective surface, that can transmit waves at a certain frequency.

**Figure 6:**
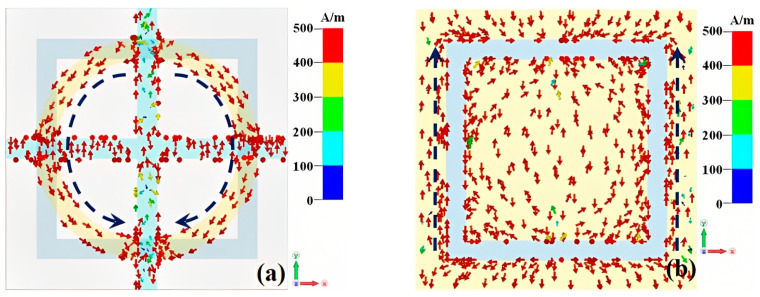
The surface current distribution of the metamaterial absorber at 8 THz when the conductivity of VO_2_ is 2 × 10^5^ S/m, (**a**) front layer; (**b**) bottom layer.

In order to further reveal the wideband absorption mechanism of the metamaterial absorber, we monitor the surface current distribution of the metamaterial absorber at 8 THz when the conductivity of VO_2_ is 2 × 10^5^ S/m, as shown in Figure 6. The surface currents on the left and right sides of the top gold ring are parallel to the downward direction, which can alternately accumulate charge at the upper and lower part of the gold split ring and thus result in electric resonance [33]. The surface current on the outer sides of the split slot at the bottom layer acts in the opposite direction with the surface current of the front gold split ring, which forms a current circuit, and accordingly produces magnetic resonance [33]. The simultaneously produced electromagnetic resonance results in the high absorption of the incident waves. The loaded resistive film allows the metamaterial absorber to function as a stable resonant circuit structure, and so its impedance matches well with the free space at a wide range near the resonant frequency and finally leads to the wideband absorption of the incident waves [34].

**Figure 7:**
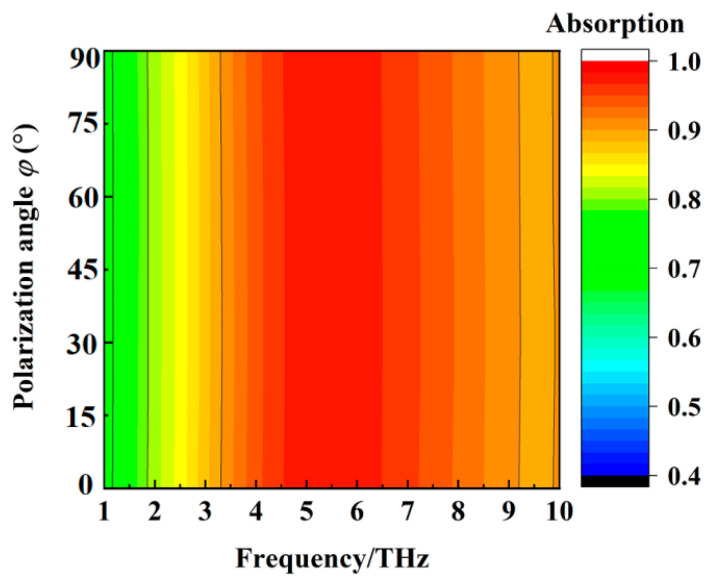
The wideband absorption property of the metamaterial absorber when the conductivity of VO_2_ film is 2 × 10^5^ S/m at different polarization angles.

**Figure 8:**
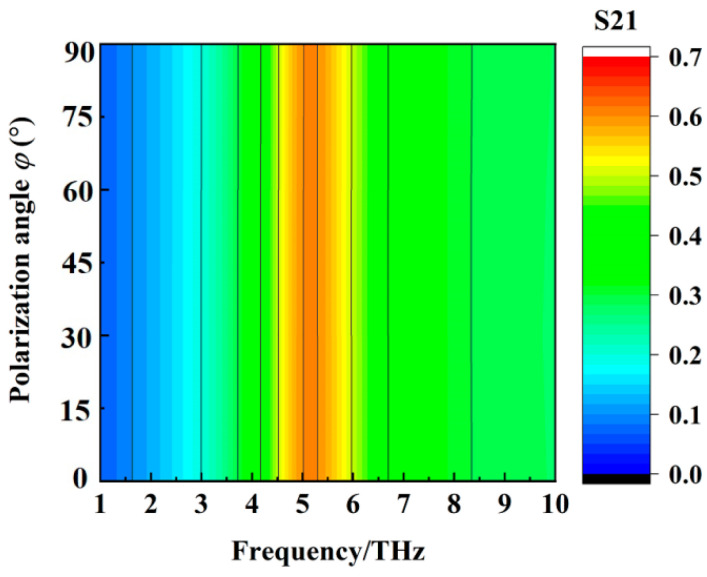
The transmission property of the metamaterial absorber when the conductivity of VO_2_ film is 10 S/m at different polarization angles.

The wideband absorption and transmission properties of the metamaterial absorber when the conductivity of VO_2_ film is 2 × 10^5^ S/m and 10 S/m at different polarization angles are shown, respectively, in Figure 7 and Figure 8. We can deduce from Figure 7 and Figure 8 that the wideband absorption and transmission properties of the metamaterial absorber are polarization−insensitive.

**Figure 9:**
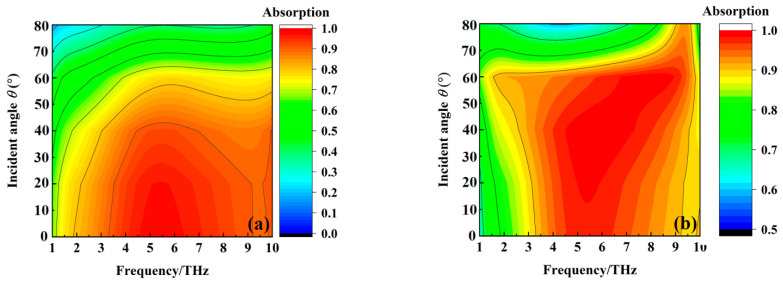
The absorption of the metamaterial absorber when the conductivity of VO_2_ film is 2 × 10^5^ S/m at different incident angles at TE and TM mode. (**a**) TE mode; and (**b**)TM mode.

**Figure 10:**
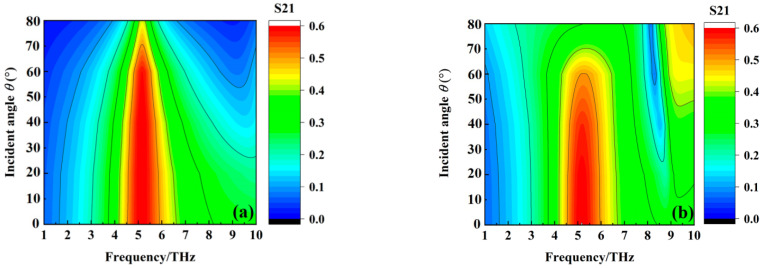
The transmission coefficient of the metamaterial absorber when the conductivity of VO_2_ film is 10 S/m at different incident angles at TE and TM mode. (**a**) TE mode; and (**b**)TM mode.

Figure 9 shows the absorption of the metamaterial absorber when the conductivity of VO_2_ film is 2 × 10^5^ S/m at different incident angles at TE and TM modes. We can see from Figure 9 that the absorption of the metamaterial absorber from 3.31 THz to 10 THz gradually decreases with the increase of the incident angles at TE mode. On the other hand, the bandwidth of the metamaterial absorber with above 90% absorption increases gradually, with the incident angle increasing from 0° to 60°, but suddenly decreases when the incident angle increases to 80° at TM mode. The absorption of the metamaterial absorber can maintain over 80% from 3.31 THz to 10 THz with the incident angle from 0° to 60°. The above results indicate that the wideband absorption property of the metamaterial absorber has the feature of wide incident angle insensitivity. Figure 10 shows the transmission of the metamaterial absorber when the conductivity of VO_2_ film is 10 S/m at different incident angles at TE and TM modes. The transmission coefficient of the metamaterial absorber at 5.15 THz gradually decreases with the increase of the incident angle at TE and TM mode, but it can maintain over 50% transmission for any incident angle from 0° to 60°. The results indicate that the transmission property of the metamaterial absorber also has the feature of wide incident angle insensitivity.

**Figure 11:**
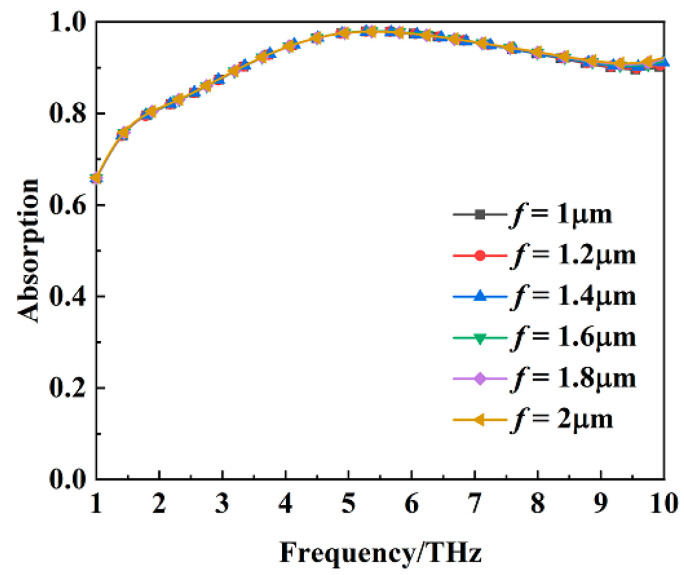
The absorption of the metamaterial absorber when the conductivity of VO_2_ film is 2 × 10^5^ S/m at different values of the structural dimension parameter *f*.

**Figure 12:**
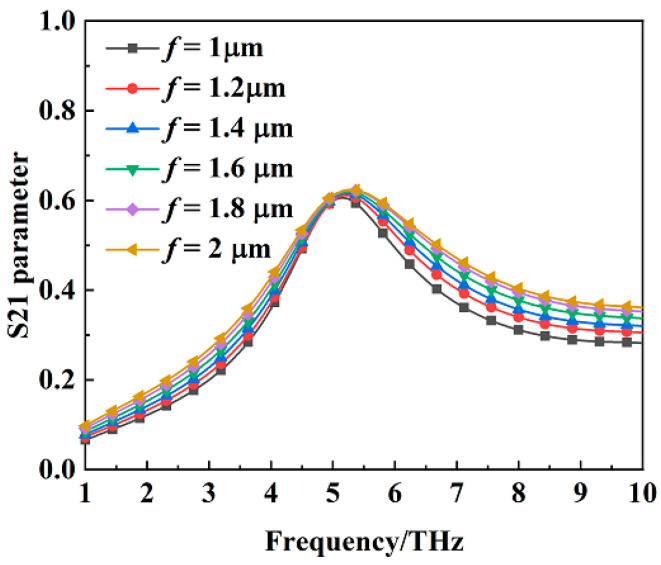
The transmission coefficient of the metamaterial absorber when the conductivity of VO_2_ film is 10 S/m at different values of the structural dimension parameter *f*.

Figure 11 shows the absorption of the metamaterial absorber when the conductivity of VO_2_ film is 2 × 10^5^ S/m at different values of the structural dimension parameter *f*. The structural dimension parameter *f* does not influence the absorption property of the metamaterial absorber. Figure 12 shows the transmission coefficient of the metamaterial absorber when the conductivity of VO_2_ film is 10 S/m at different values of the structural dimension parameter *f*. The transmission coefficient at 5.15 THz is basically the same at different values of the structural dimension parameter *f*.

## 4. Conclusions

In this paper, we present a thermally controlled multifunctional metamaterial absorber with switchable wideband absorption and transmission at the THz band, which is based on the thermally controlled conductivity of VO_2_ film. By controlling the temperature of VO_2_ thin film, switchable functionality between wideband absorption and transmission can be realized. We demonstrated that the wideband absorption and transmission property of the metamaterial absorber is polarization insensitive and has the feature of wide incident angle insensitivity. It has great potential application value in the fields of stealth of communication equipment and radar at the THz band.

## Data Availability

The data that support the findings of this study are available from the corresponding author upon reasonable request.
